# Vagus Nerve Stimulation in Movement Disorders, from Principles to a Systematic Review of Evidence

**DOI:** 10.1002/mds.70044

**Published:** 2025-09-30

**Authors:** Francesca Proietti, Matteo Maria Ottaviani, Elisabetta Burchi, Giorgio Vivacqua, Gaia Anzini, Riccardo Antonio Ricciuti, Fioravante Capone, Vaughan G. Macefield, Vincenzo Di Lazzaro, Massimo Marano

**Affiliations:** ^1^ Unit of Neurology, Neurophysiology, Neurobiology and Psychiatry, Department of Medicine University Campus Bio‐Medico of Rome Rome Italy; ^2^ Department of Neurosurgery Università Politecnica delle Marche Ancona Italy; ^3^ Department of Neurosurgery Azienda Ospedaliera di Perugia Perugia Italy; ^4^ Scuola Superiore di Scienze dell'Educazione “San Giovanni Bosco”, aggregata alla Facoltà di Scienze dell'Educazione Università Pontificia Salesiana Rome Italy; ^5^ Parasym London United Kingdom; ^6^ Department of Microscopic and Ultrastructural Anatomy Campus Biomedico University of Roma Rome Italy; ^7^ Fondazione Policlinico Universitario Campus Bio‐Medico Rome Italy; ^8^ UOC Neurosurgery Azienda Ospedaliera San Camillo Forlanini Rome Italy; ^9^ Department of Neuroscience, School of Translational Medicine Monash University Melbourne Victoria Australia

**Keywords:** cervical dystonia, essential tremor, movement disorders, neuromodulation, Parkinson's disease, transcutaneous stimulation, vagus nerve stimulation

## Abstract

**Background:**

The vagus nerve (VN), the principal component of the parasympathetic branch of the autonomic nervous system (ANS), mediates bidirec communication between the central nervous system (CNS) and peripheral organs. Vagus nerve stimulation (VNS), delivered through invasive (iVNS) or non‐invasive (transcutaneous cervical [tcVNS] and transcutaneous auricular [taVNS]), exerts multimodal effects on multiple circuits and neurotransmitters, and is currently under investigation in movement disorders.

**Objectives:**

This systematic review evaluates the therapeutic potential of VNS in movement disorders (MDs), with a focus on Parkinson's disease (PD), parkinsonism, tremor and essential tremor (ET), cervical dystonia (CD), and Tourette's syndrome (TS).

**Methods:**

A systematic search of PubMed, Web of Science, and Scopus was conducted for studies published between 2020 and 2025, following Preferred Reporting Items for Systematic Reviews and Meta‐Analyses 2020 guidelines.

**Results:**

Thirty‐two studies met the inclusion criteria: 22 on PD or parkinsonism (14 clinical and 8 preclinical on PD, 1 clinical on multisystem atrophy), 6 on tremor and ET (4 clinical, 2 preclinical), 2 clinical studies on CD, and 1 clinical study on TS. Across conditions, VNS was reported to improve motor and non‐motor, enhance synaptic plasticity, modulate neurotransmitter activity relevant to MDs (GABA, norepinephrine, dopamine, and acetylcholine), and reduce neuroinflammation.

**Conclusions:**

Current evidence supports the multimodal effect of VNS in MDs, particularly in PD, where the most consistent benefits were observed. Non‐invasive taVNS represents a promising, safer alternative to iVNS. Larger randomized controlled trials with standardized protocols are needed to validate efficacy, optimize stimulation parameters, and determine long‐term clinical and biological impact. © 2025 The Author(s). *Movement Disorders* published by Wiley Periodicals LLC on behalf of International Parkinson and Movement Disorder Society.

Vagus nerve stimulation (VNS) is increasingly recognized as an innovative experimental therapeutic intervention for movement disorders (MDs), reflecting the vagus nerve (VN) regulatory role in motor control and its involvement in neurodegenerative disease pathophysiology, including Parkinson's disease (PD).^1^ Afferent fibers of the VN convey sensory input to the nucleus of the solitary tract (NTS), which projects to multiple structures including the locus coeruleus (LC), dorsal raphe nucleus (DRN), parabrachial nucleus (PBN), pedunculopontine nucleus (PPN), basal forebrain, and thalamus.^2^, ^3^, ^4^, ^5^ These connections underscore the role of the VN in modulating circuits involved in movement, mood, and learning. VNS has been shown to modulate neural networks and promote synaptic plasticity, supporting its potential as a therapeutic strategy for MDs such as PD, tremor, and dystonia.^6^ Stimulation can be delivered invasively via cervical implants (invasive VNS [iVNS]) or non‐invasively through transcutaneous cervical (tcVNS) or transcutaneous auricular (taVNS) stimulation. iVNS received United States Food and Drug Administration (FDA) approval for drug‐resistant epilepsy in 1994^7^, ^8^, ^9^, ^10^, ^11^ and for treatment‐resistant depression in 2005.^12^, ^13^, ^14^, ^15^, ^16^, ^17^, ^18^ Recent advances in non‐invasive approaches have increased the accessibility and feasibility of VNS for long‐term use, broadening its therapeutic application in neurological and psychiatric disorders. This systematic review examines recent advances in the application of VNS in MDs, including parkinsonism, tremor, dystonia, and tics. The findings are considered in the context of VN anatomy, VNS techniques, and clinical applications, with particular emphasis on optimizing stimulation protocols.

The subsequent section details the methodology and results, integrating preclinical and clinical evidence to delineate the current landscape of VNS‐based therapies for MDs.

## Subjects and Methods

We conducted a systematic search of PubMed, Web of Science, and Scopus, for articles published between 2020 and 2025. The search strategy used the following terms: (“vagus nerve stimulation”[Title/Abstract]) AND (“parkinson's disease”[Title/Abstract] OR “tremor”[Title/Abstract] OR “dystonia”[Title/Abstract] OR “movement disorder”[Title/Abstract] OR “parkinsonism”[Title/Abstract]); (“vagus nerve stimulation” AND [“dystonia” OR “tremor” OR “parkinson's disease” OR “movement disorders” OR “parkinsonism”]).

Studies were eligible if they investigated the effects of VNS on motor or non‐motor symptoms in adult patients or animal models of MDs. Eligible study designs included randomized controlled trials (RCTs), preclinical experimental studies, observational studies, case reports, and case series. Protocols, conference abstracts, review articles, opinion papers, and editorials were excluded. This review was conducted in accordance with the Preferred Reporting Items for Systematic Reviews and Meta‐Analyses (PRISMA) guidelines. The methodological quality of the included RCTs was assessed using the Cochrane Risk‐of‐Bias 2.0 tool (Table S1).^19^

## Results

**FIG. 1:**
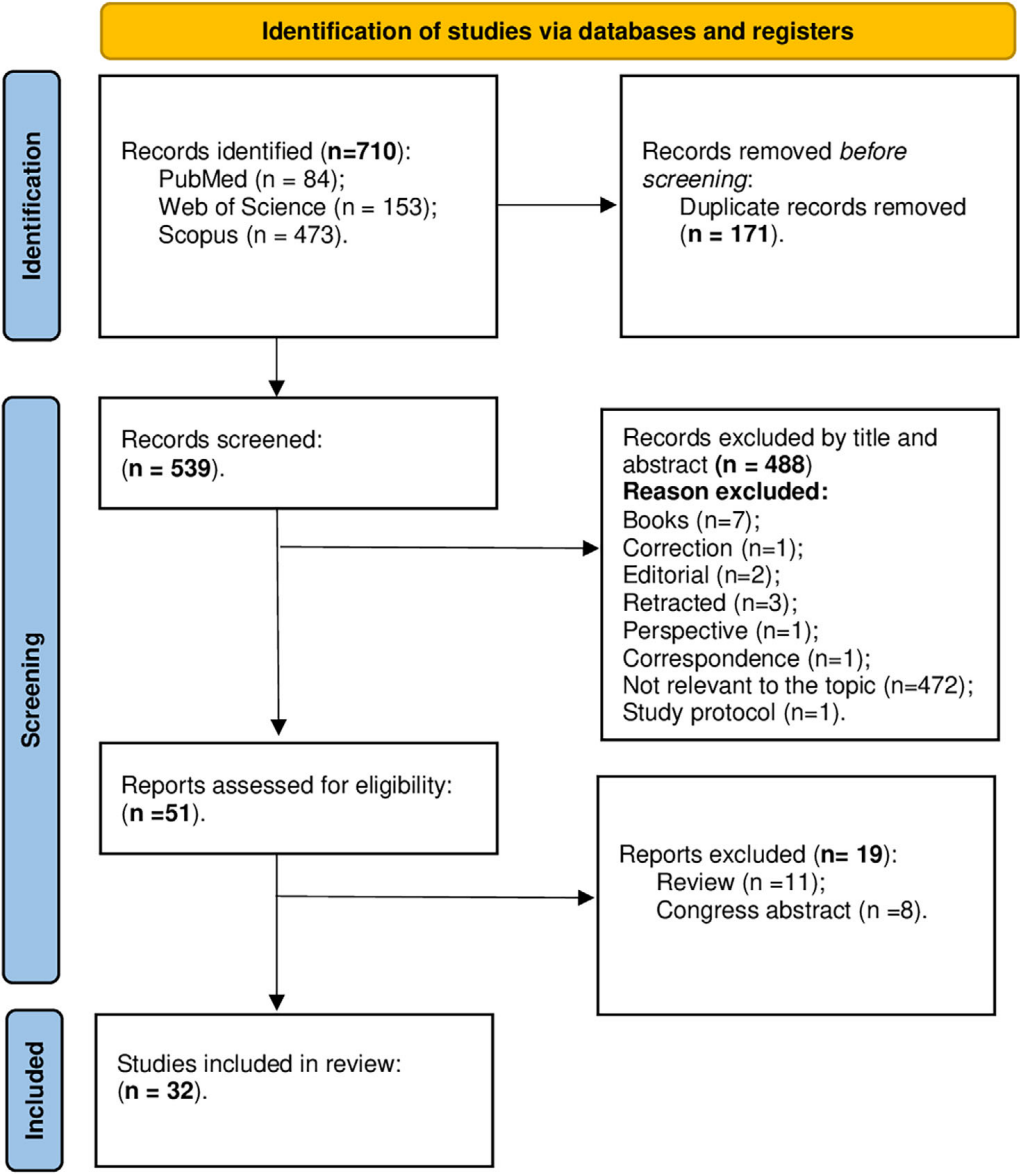
Preferred Reporting Items for Systematic Reviews and Meta‐Analyses (PRISMA) flow chart of screening and selection. [Color figure can be viewed at wileyonlinelibrary.com]

A total of 710 records were identified through database searches (PubMed = 84; Web of Science = 153; Scopus = 473). After removal of 171 duplicates, 539 records were screened by title and abstract. Of these, 488 were excluded because of irrelevance to the topic (n = 472), or because they comprised book chapters, editorials, retracted articles, perspectives, correspondence, study protocols, or other non‐eligible formats. The remaining 51 full‐text articles were assessed for eligibility, of which 19 were excluded (11 reviews and 8 congress abstracts). Ultimately, 32 studies were included in the review (Fig. 1), encompassing both preclinical and clinical research on VNS in MDs. Studies are organized chronologically and categorized by condition—PD and parkinsonisms, tremor and essential tremor (ET), dystonia and tics—and by study type (clinical vs. preclinical) to highlight research progression and the growing interest in VNS applications for MDs. No results were found for other MDs such as chorea and functional MDs. Myoclonus were treated in the context of epilepsy, therefore, it was not included.

Specifically, we identified 14 clinical and eight preclinical studies on PD and one clinical study on atypical parkinsonism (multisystem atrophy type C [MSA‐C]), four clinical and two preclinical studies on tremor and ET, two clinical studies on cervical dystonia (CD), one clinical study on Tourette syndrome (TS). The key characteristic and findings of these studies are summarized in the accompanying tables.

### VNS in PD and Parkinsonisms: Preclinical Experiments

In 2017, Farrand and colleagues^20^ investigated the effects of iVNS on locomotion, neuronal survival, and neuroinflammation using a double‐lesion rat model that targeting both the LC noradrenergic (NE) and substantia nigra (SN) dopaminergic (DA) systems. Their findings demonstrated that iVNS improved locomotion, reduced ɑ‐synuclein in SN tyrosine hydroxylase (TH) neurons, promoted cell survival, and attenuate glial activation.^20^ Building on this work, Jiang and collaborators^21^ examined taVNS in a 6‐OHDA rat model in 2018, reporting improved motor performance, increased TH expression, and reduced proinflammatory cytokines (TNF‐ɑ, IL‐1β) in the SN. Moreover, taVNS enhanced regulatory T‐cell activity while suppressing pro‐inflammatory T‐helper cells, underscoring its potential role in immune modulation.^21^ In 2019, Farrand et al.^22^ investigated the functional involvement of the brain‐derived neurotrophic factor (BDNF)‐TrkB signaling pathway in mediating the effect of iVNS in a double‐lesion rat model of PD. They found that iVNS improved locomotor activity, promoted DA neuron survival, and reduced neuroinflammation. However, pharmacological blockade of TrkB with ANA‐12 partially attenuated the neuroprotective and anti‐inflammatory effects of VNS, while locomotor improvements remained unaffected.^22^ In 2020, the same group investigated the differential effects of three iVNS (LivaNova) paradigms: low‐frequency (systemic anti‐inflammatory standard; 10 Hz; 250 μs pulse width; 0.75 mA intensity; 30 minutes/24 hours), standard‐frequency (epilepsy clinical standard; 20 Hz; 250 μs; 0.75 mA; 30 seconds ON/5 minutes OFF) and high‐frequency (biomimetic experimental microburst therapy; 300 Hz; 250 μs; 0.75 mA; 24/24 hours) stimulation for 10 days. Using a two‐step lesion rat model of PD, animals were randomly assigned to treatment groups receiving one of the stimulation protocols.^23^ Although both standard and burst iVNS increased locomotion, high‐frequency microburst iVNS yielded the most robust therapeutic effects, including greater motor improvement, enhanced NE release in the prefrontal cortex (PFC), increased TH neuron survival, reduced ɑ‐synuclein, restored NE and DA levels, and reduced neuroinflammation.^23^ In 2021, Kin and colleagues^24^ investigated the effects of different stimulation intensities (0.1 mA; 0.25 mA; 0.5 mA; and 1 mA; control group with no VNS) using fixed parameters (30 Hz; 500 μs; 30 seconds ON/5 minutes OFF) in 6‐OHDA rat model of PD. Stimulation at 0.25 to 0.5 mA significantly improved motor behavior, preserved DA and NE neurons, and reduced astrocyte and microglial activation.^24^ Although most studies have focused on the left‐sided stimulation, recent evidence suggested that the right VN has stronger projections to midbrain DA neurons, making it a promising target for PD.^24^ In 2022, Wang and colleagues^25^ investigated the therapeutic effect of right‐sided iVNS in a rotenone‐induced rat model of PD. Stimulation was delivered for 14 days using standard parameters (30 Hz; 500 μs; 1 mA; 30 seconds ON/5 minutes OFF). Right iVNS significantly improved motor function, as demonstrated by increased locomotion/resting activity in the open‐field test. In parallel, right iVNS upregulated TH‐expression, preserved DA neurons in the SN and the ventral tegmental area (VTA), reduced ɑ‐synuclein aggregation, and enhanced vesicular monoamine transporter 2 (VMAT2) expression, a key protein for DA storage and release.^25^ Hosomoto and colleagues^26^ investigated the effects of continuous and selective iVNS in a rat PD model, specifically comparing afferent or efferent pathways. Selectivity was achieved by performing targeted vagotomy, severing either the afferent or the efferent branch of the VN, thereby restricting stimulation to the remaining intact fibers. Fifty‐one rats were allocated to five groups: intact iVNS, afferent iVNS (left caudal vagotomy), efferent iVNS (left rostral vagotomy), sham stimulation, and vagotomy alone. The results demonstrated that iVNS exerted therapeutic effects in PD rats. Selective afferent iVNS (i.e., stimulation after caudal vagotomy) preserved DA neurons and increased DA β‐hydroxylase density, whereas efferent iVNS (i.e., stimulation after rostral vagotomy) conferred no benefit. Importantly, vagotomy alone did not improve outcomes, indicating that the beneficial effects were specifically mediated by afferent‐selective iVNS.^26^ Findings were corroborated by Cheng et al.,^27^ who tested different stimulation intensities in a rat model of PD using implanted electrodes targeting the cervical VN. iVNS significantly improved motor performance and NE c‐FOS activity in LC, reduced TNF‐ɑ, IL‐1β, Iba‐1, GFAP, and enhanced DA neuron survival, further supporting its potential as a neuroprotective therapy for PD.^27^ A summary of preclinical studies is provided in Table 1.

**TABLE 1:** Preclinical PD and tremor studies

Author, y	Study design model	Technique	VNS parameters	Intervention duration	Outcome measures	Main results	Strengths	Limitations
Preclinical PD								
Farrand et al., 2017^20^	Interventional, randomized, controlled DSP‐4 + 6‐OHDA dual lesion rats	iVNS	I: 0.8 mA PW: 100 μs F: 30 Hz DC: 500 ms train (15 pulses) every 30 s WF: biphasic	2 × 30 min/day 10 days	Locomotion (distance traveled, Digiscan photobeam) LC‐NE neurons SN‐DA neurons ɑ‐synuclein neuroinflammation	Improved locomotion in lesioned rats Increased TH expression in striatum, SN, and LC Reduced α‐synuclein accumulation in SN TH+ neurons Decreased astrocytic and microglial activation	Progressive dual‐lesion NE/DA PD model; multiple behavioral and histological outcome measures	Preclinical dual‐lesion model hypothesis driven, may not recapitulate PD pathology; short observation period; behavioral outcomes restricted mainly to locomotion.
Jiang et al., 2018^21^	Interventional, randomized, controlled	taVNS	I: 0.8 mA PW: 500 μs F: 30 Hz DC: 500 ms ON/30 s OFF	4 × 30 min 30 min each other day for 8 days	Motor tests: rotarod, beam‐walking, apomorphine‐induced rotation TH+ neuron survival in SNpc Treg/Th17 cell ratio α7 nAChR expression (ventral midbrain) Cytokines: TNF‐α, IL‐1β	Improved motor performance (rotarod, beam‐walking, reduced rotations) Increased survival of SN TH+ neurons Increased α7 nAChR expression Reduced TNF‐α and IL‐1β expression Immunomodulation: enhanced Treg cells, reduced Th17 cells expression	Validated unilateral 6‐OHDA PD model; integrated behavioral, histological and molecular assessments	Animal model; limited duration (8 days); behavioral tests restricted to locomotion/coordination, no cognitive or affective outcomes.
Farrand et al., 2019^22^	Interventional, randomized, controlled DSP‐4 + 6‐OHDA dual lesion rats	iVNS	I: 0.8 mA PW: 100 μs F: 30 Hz DC: 500 ms ON/30 s OFF WF: biphasic	2 × 30 min/day 10 days	Locomotion (Digiscan photobeam) BDNF/TrkB pathway activation (Western blot, immunohistochemistry) ɑ‐synuclein density in SNpc TH+ neurons Neuroinflammatory markers	VNS neuroprotection associated with BDNF–TrkB signaling Reduced α‐synuclein accumulation and neuroinflammation;	First study to examine BDNF–TrkB signaling in VNS‐induced neuroprotection in PD; comprehensive behavioral and molecular analyses	Short‐term preclinical model; TrkB inhibition did not abolish locomotor benefit, suggesting additional mechanisms not explored.
Farrand et al., 2020^23^	Interventional, randomized, controlled DSP‐4 + 6‐OHDA dual lesion rats	iVNS (LivaNova)	I: 0.75 mA F1: 10 Hz F2:20 Hz F3: 300 Hz PW: 250 μs DC1: 30 min ON/23.5 h OFF DC2: 30s on/5 min DC3: 19 s interbust interval 24 h/24 h WF:	10 days	Locomotor activity (Digiscan photobeam) Motor coordination (cylinder test) TH+ neuron survival in SNpc and striatum α‐synuclein expression Neuroinflammation (GFAP, LC‐NE, SN‐DA markers)	Standard and burst taVNS improved locomotor performance Burst taVNS showed the strongest neuroprotective effect: increased TH+ neurons, restored NE and DA levels, reduced α‐synuclein, and decreased neuroinflammation	Direct comparison of multiple stimulation paradigms (low, standard, burst) in the same validated PD model	Preclinical study; limited duration; mechanistic pathways underlying burst vs. standard taVNS not fully elucidated.
Kin et al., 2021^24^	Interventional, randomized, controlled (single blind)	iVNS	I: 0.1/0.25/0.5/1 mA PW: 500 μs F: 30 Hz DC: 30 s ON/5 min OFF	14 days	Motor: Cylinder test, methamphetamine‐induced rotation test Neuronal survival: TH+ dopaminergic neurons in SNpc, DBH+ noradrenergic neurons in LC Neuroinflammation: Iba‐1, GFAP	0.25 and 0.5 mA VNS: Behavioral improvements Preserved TH+ dopaminergic neurons Increased DBH+ noradrenergic neurons in LC Reduced neuroinflammation (astrocytic and microglial activation)	Direct comparison of multiple stimulation intensities	Animal model; short duration (14 days); limited exploration of long‐term neuroprotective effects.
Wang et al., 2022^25^	Interventional, randomized, controlled (single blind)	iVNS (right‐sided implanted)	I: 1 mA PW: 500 μs F: 30 Hz DC: 30 s ON/5 min OFF WF: –	14 days	Open field test TH ɑ‐synuclein expression VMAT2 expression	Improved motor function Neuroprotective effects, increasing TH and VMAT2 expression while reducing α‐synuclein accumulation in the SN and VTA	Right cervical VNS; combined behavioral and neurobiological outcome measures	Preclinical study; right‐sided stimulation effects on broader circuits not fully clarified; potential autonomic/cardiac side effects not assessed.
Hosomoto et al., 2023^26^	Interventional, randomized, controlled (single blind)	iVNS	I: 0.1/0.25 mA PW: 500 μs F: 30 Hz DC: 30 s ON/5 min OFF WF: –	14 days	Motor: Cylinder test, Methamphetamine‐induced rotation test TH‐immunostaining (SNpc) neuroinflammation: Iba‐1, GFAP staining DBH staining (LC)	Improved motor function Preserved TH+ dopaminergic neurons in SNpc Reduced microglial activation and astrocytic activation Increased noradrenergic neuron density in LC	First study testing continuous VNS stimulation in PD model; highlighted role of afferent LC‐NA neurons in mediating VNS neuroprotection; comprehensive biomarker evaluation (dopaminergic, noradrenergic, inflammatory)	Single‐lesion model; short duration; no electrophysiological validation; long‐term outcomes not evaluated.
Cheng et al., 2024^27^	Interventional, randomized, controlled	iVNS	I: 0.8 mA PW: 250 μs F: 30 Hz DC: 1 s ON/29 s OFF WF: –	11 days	Motor: Pole test; Rotarod test; open field test DA neurons: TH staining, DAPI, PCR c‐FOS activity in LC Neuroinflammation: Iba‐1, GFAP, TNF‐α, and IL‐1β	Improved motor function Attenuated dopaminergic neuronal loss Increased LC‐NE neuronal activity Reduced neuroinflammation	First study testing low‐charge VNS protocol in a PD model; demonstrated therapeutic benefits while minimizing stimulation side effects; identified a role for LC‐NA neurons in mediating neuroprotection	Preclinical animal model; unilateral lesion.
Preclinical ET								
Handforth et al., 2001^43^	Interventional, open label Harmaline (rats)	iVNS	I: 0.5 mA PW: 500 μs F: 20 Hz DC: 5 min ON/10 min OFF WF: biphasic	5 × 5 min	Physiograph recordings Blinded scoring of tremor amplitude.	Reduction of harmaline‐induced tremor Effect duration: 3 min (baseline within 10 min) No habituation	Objective measurement; blinded videorating; multiple trials	Preclinical harmaline‐induced tremor model; acute effects only; unclear specificity to tremor vs. general motor suppression.
Krahl et al., 2004^44^	Interventional, randomized, controlled (single blind) ‐ Harmaline (rats)	iVNS	I: 0.5 mA PW: 500 μs F: 20 Hz DC: 10 min ON WF: –	10 min	Tremor monitor chamber Blinded scoring of tremor amplitude.	Reduction of harmaline‐induced tremor by 40% Tremor suppression confirmed by blinded assessment	Objective measurement; blinded assessment	Animal model; acute single‐session study; mechanism of tremor suppression not established; only partial tremor reduction observed.

Abbreviations: PD, Parkinson's disease; VNS, vagus nerve stimulation; iVNS, surgically implanted vagus nerve stimulation; I, current intensity; F, frequency; DC, duty cycle; WF: waveform; PW, pulse width; LC‐NE, locus coeruleus noradrenergic neurons; SN‐DA, substantia nigra dopaminergic neurons; TH, tyrosine hydroxylase; SN, substantia nigra; DA, dopaminergic; C, control/sham stimulation; taVNS, transcutaneous auricular vagus nerve stimulation; SNpc, substantia nigra pars compacta; α7 nAChRs, α7 nicotinic acetylcholine receptors; DBH, dopamine β hydroxylase; DAPI, 4,6‐diamino‐2‐phenyl indol; GFAP, glial fibrillary acidic protein; IO, inferior olive; VTA, ventral tegmental area; PCR, polymerase chain reaction; ET, essential tremor.

### VNS in Parkinson's Disease and Parkinsonism: Clinical Experiments

Bokkala‐Pinniti and colleagues were the first to report clinical benefits of iVNS in PD, describing a 64‐year‐old woman with mild PD and drug‐resistant epilepsy who exhibited a sustained 25% improvement in parkinsonian symptoms following iVNS (30 Hz, 250 μs, 1.5 mA, 30 seconds ON/3 minutes OFF).^28^ Nearly a decade later, Mondal et al.^29^ conducted an open‐label study in 19 PD patients (12 with freezing of gait [FOG]) using left tcVNS (GammaCore, ElectroCore, NJ, USA), reporting improvement in gait speed, step length, turning, and Unified Parkinson's Disease Rating Scale (UPDRS) III scores. These results were subsequently confirmed in the sham‐controlled trial with 30 patients by Morris et al.,^30^ where only the active stimulation group demonstrated reduced step time and step length variability as assessed with an instrumented walkway. Although the precise underlying mechanism remained unclear, Torrecillos et al.^31^ provided evidence that left tcVNS modulates β‐band (13–30 Hz) activity in the subthalamic nucleus (STN), a known biomarker of PD. They observed a cumulative reduction in STN β power during four consecutive 100‐s stimulations at 25 Hz.^31^ Gait improvements with left taVNS were further corroborated by Marano and colleagues,^33^ who demonstrated enhanced step length and reaction times in both *on* and *off* DA medication states using a digital 10‐meter Timed‐Up‐and‐Go (TUG) test after a 30‐min taVNS session (30 seconds trains at 20 Hz, 300 μs).^32^ In their second crossover study, Marano et al^33^ applied real and sham taVNS to 10 PD patients with the Percept deep brain stimulation (DBS) system (Medtronic, Illinois), enabling direct STN β power recordings. Only real stimulation reduced β power and improved gait, reinforcing the hypothesis that taVNS acts through modulation of pathological oscillations.^33^

Van Midden et al.^34^ investigated frequency‐dependent effects of taVNS (Jan Slapsak s.p. stimulator, Jesenice, Slovenia, connected to Nemos electrodes) on gait in 30 advanced PD patients using a crossover design comparing 25 Hz, 100 Hz, and sham stimulation. taVNS at 100 Hz improved stride length and arm swing velocity, decreasing anticipatory postural adjustments (APA) duration, whereas 25 Hz improved gait speed, stride length, decreasing turn duration.^34^

To further elucidate neural mechanisms of taVNS in PD, Fu et al.^35^ conducted a crossover resting‐state functional magnetic resonance imaging (fMRI) study in 50 PD patients across three conditions: baseline (no stimulation), real and sham taVNS (SDZ‐IIB, Hwato, Suzhou, China). Real taVNS induced a significant decrease in the amplitude of low frequency fluctuations (ALFF) in several right‐hemisphere regions—including the superior parietal lobule (SPL), precentral and postcentral gyrus, middle occipital gyrus and cuneus—compared to both baseline and sham.^35^ Conversely, baseline ALFF values in the right SPL negatively correlated with Unified Parkinson's Disease Rating Scale (UPDRS) III motor scores and quality of life.

Multi‐session taVNS has been evaluated in several studies, including investigations aimed at elucidating the neurobiological mechanisms underlying VN stimulation. Zhang et al.^36^ evaluated gait and cortical activity in 22 PD patients and 14 controls in a randomized study. After 7 days of twice‐daily left‐ear taVNS (tVNS501, RISHENA, Changzhou, China), gait analysis of 5‐meter TUG tests revealed improvement in stride velocity and step length.^36^ Concurrent functional near‐infrared spectroscopy (fNIRS) recordings showed reduced activity in the left primary somatosensory cortex, consistent with enhanced sensorimotor integration.^36^ Psychological effects of taVNS were further investigated in a large study of 90 PD patients (with and without anxiety) and 30 healthy controls.^37^ After 14 days of daily stimulation, anxiety (Hamilton rating scale for Anxiety [HAM‐A]) and UPDRS I–III scores improved, whereas fNIRS demonstrated increased oxyhemoglobin levels in the left inferior frontal gyrus, correlating with anxiety relief and supporting cortical modulation by taVNS (tVNS501).^37^ Mondal et al.^38^ extended these findings in a crossover study with 33 PD patients with FOG, showing that 1 month daily self‐administered tcVNS (two 120 second GammaCore stimulation at intervals of 5–10 minutes) improved gait performance, reduced TNF‐α, and increased BDNF and glutathione, without major adverse events.

The effect of multi‐session taVNS was also explored by Lench et al.^39^ with a double‐blind, sham‐controlled RCT in 30 patients with mild‐to‐moderate PD. Although no significant changes were detected at the group‐level in Movement Disorder Society (MDS)‐UPDRS motor scores and self‐reported outcomes, a subgroup of patients exhibited improvements in bradykinesia and tremor. The latter was investigated by Menekseoglu et al^40^ in a small before‐after study including five patients treated with taVNS (Vagustim TENS). Stimulation was applied to the left, right, or both ears in separate sessions (20 minutes each; 0.8 mA, 10 Hz, 300 μs). A reduction in right hand resting tremor amplitude, measured via a smartphone application, was observed after bilateral application, although no significant changes emerged on clinical examination. Left side application reduced the sympathetic nervous system (SNS) index at heart rate variability (HRV) analysis. Beyond motor and behavioral outcomes, Kaut et al.^41^ assessed gastrointestinal effects in 19 PD randomized patients, reporting significant improvements in Gastrointestinal Symptom Rating Scale scores after 4 weeks of four daily tcVNS (GammaCore) applications, although 13C breath tests revealed minimal impact on gastric motility. Details of the clinical experimental studies are summarized in Table 2.

**TABLE 2:** Clinical studies on PD, tremor, and ET

Author, y	Study design, participant num medication state	Technique (commercial name) target sham modality	VNS parameters	Intervention duration	Outcome measures	Main results	Strengths	Limitations
Clinical studies in PD								
Single session VNS interventions								
Mondal et al., 2019^29^	Interventional, open label n = 19 (12 with FOG) Med: N/A (presumably OFF DOPA)	tcVNS (GammaCore) Left neck VN	I: – PW: 100 μs F: 25 Hz DC: – WF: 5 kHz sine	2 × 120 s, 15‐min interval	UPDRS III Gait analysis Videotapes (FOG)	Improvement of UPDRS III Reduction of step count and stride variability, increased stride velocity and step and stride length Reduced rotation step number of FOG patients	Objective gait assessment (GAITRite); Blinded video rating of FOG	Small heterogeneous cohort, open‐label design
Morris et al., 2019^30^	Interventional, randomized, sham‐controlled (double blind) n = 29 Med: ON DOPA	tcVNS (GammaCore) Left neck VN C: manufacturer sham device	I: – F: 25 Hz PW: 100 μs F: 25 Hz DC: – WF: 5 kHz sine	1 × 120 s	Gait analysis	Decrease step length variability	Sham controlled design; Objective gait assessment (instrumented walkway)	Small cohort
Marano et al., 2022^32^	Interventional, randomized sham‐controlled, crossover (double‐blind) n = 12 Med: ON DOPA	taVNS (custom) Left inner tragus ABVN C: earlobe	I: – PW: 300 μs F: 20 Hz DC: 30 s ON/4.5 min OFF WF: continuous	6 × 30 s, 4.5 min intervals (30 min total)	Gait analysis (TUG) Flanker test UPDRS III VAS	Increased stride length, swing amplitude, speed and reduced total TUG time Reduced reaction times at Flanker test	Objective measurement adopted for gait and cognition (Mon4t TUG, and Flanker test) Double‐blind	Small sample size; self‐controlled
Torrecillos et al., 2022^31^	Interventional, open label n = 7 Med: OFF DOPA	tcVNS (GammaCore) Left neck VN	I: – F: 25 Hz PW: 100 μs F: 25 Hz DC: – WF: 5 kHz sine	4 × 100 s (120 s intervals)	STN‐LFPs	Bilateral reduction in STN low‐β oscillation power	First direct demonstration that tcVNS modulates pathological STN β oscillations in PD	Small sample size; uncontrolled
Menekseoglu et al., 2023^40^	Interventional, crossover, (double‐blind) n = 5 Med: OFF DOPA	taVNS (Vagustim TENS) Inner tragus and cymba conchae ABVN	I: 0.8 mA PW: 300 μs F: 10 Hz DC: – WF: –	3 × 20 min 1 × left 1 × right 1 × bilat (1 per day)	Tremor analysis UPDRS III HRV	Reduced right hand tremor after bilat. taVNS Reduced SNS index after left taVNS	Use of objective assessment of tremor (G‐sensor Logger) and HRV (Kubios HRV)	Small sample size; uncontrolled
Marano et al., 2024^33^	Interventional, randomized sham‐controlled, crossover (double‐blind) n = 10 Med: OFF DOPA	taVNS (custom) Left inner tragus ABVN C: earlobe	I: – PW: 300 μs F: 25 Hz DC: 120 s ON/60 s OFF WF: continuous	4 × 120 s ON/60 s OFF	Gait analysis (TUG) UPDRS III STN LFPs	Increased walking speed Reduced gait variability and TUG time Reduced right STN β power Correlation between β power reduction and walking speed improvement	First study to demonstrate direct interaction between taVNS, STN oscillatory activity and gait parameters	Small sample size, self‐controlled
Van Midden et al., 2024^34^	Interventional, randomized, sham‐controlled (double‐blind) n = 30 Med: ON DOPA	taVNS (Nemos electrode + Jan Slapsak s.p. stimulator) Left cymba conchae ABVN C: earlobe	I: – PW: 300 μs F: 25 Hz/100 Hz DC: – WF: –	Single session with multiple trials	Gait analysis	Improved arm swing velocity, stride length increased APA duration (100 Hz) Improved stride length, gait speed, double 360° turn (25 Hz)	Objective gait assessment with IMUs (iSAW, d360°t, Mobility Lab software); different frequencies comparison.	Small sample size, single session, possible carryover effect.
Fu et al., 2024^35^	Interventional, randomized, sham‐controlled, crossover (single‐blind) n = 50 Med: OFF DOPA	taVNS (Hwato) Left cymba conchae C: left tail of the helix	I: 1 to 2.5 mA PW: 200 μs F:20/100 Hz DC: – WF: –	8 min	UPDRS PDQ NMSS MMSE fMRI (ALFF)	taVNS was associated with lower ALFF in motor‐related areas (PreCG, PoCG, SPL) A negative correlation was observed between ALFF under real stimulation and PD severity (UPDRS III, PDQ scores)	taVNS influences brain activity in PD, particularly in motor and visual processing networks; explores lateralization effects of taVNS	Uncontrolled; focused only on the off medication state; short‐term effects of taVNS; no long term follow up; unclear correlation between clinical parameters and ALFF
Multi‐session/chronic VNS interventions								
Bokkala‐Pinniti et al., 2008^28^	Observational, single‐case	iVNS	I: 1.5/2.0 mA PW: 250 μs F: 30 Hz DC: 30 s ON/3 min OFF WF: –	Continuous over 6 months	Seizure frequency UPDRS III	Improvement in seizure control and Parkinsonian symptoms (resting tremor and bradykinesia)	Long term follow‐up	Single case study, uncontrolled
Kaut et al., 2019^41^	Interventional, randomized, sham‐controlled (double‐blind) n = 19	tcVNS (GammaCore) Neck VN C: manufacturer sham device	I: – F: 25 Hz PW: 100 μs F: 25 Hz DC: – WF: 5 kHz sine	4 × 120 s/day 4 weeks	GSRS 13C breath test	Improvement in GSRS	Objective assessment of gastrointestinal function.	Small sample size; lack of objective gastrointestinal motility changes (13C‐labeled breath test); indirect assessment of gastrointestinal motility
Zhang et al., 2023^36^	Interventional, randomized, sham‐controlled (double blind) n = 22	taVNS Left cymba conchae ABVN C: earlobe	I: – PW: 500 μs F: 20 Hz DC: 60 s ON/10 s OFF WF: –	2 × 30 min/day for 7 days	UPDRS III TUG Tinetti scale fNIRS (ΔHbO₂ in left S1)	Improved step length, stride velocity, stride length, step length variability, gait cycle, and double support time Decreased ΔHbO₂ in the left primary somatosensory cortex	Integration of behavioral and neurophysiological measures (fNIRS); neural mechanism insights: provides evidence that taVNS remodels sensorimotor integration	Small sample size, short follow‐up; no significant improvement in global clinical scales; fNIRS limitations in detecting subcortical structures
Lench et al., 2023^39^	Interventional, randomized, sham‐controlled (double blind) n = 30	taVNS (custom) Inner tragus ABVN C: earlobe	I: 200% perceptual threshold PW: 500 μs F: 25 Hz DC: 60 s ON/30 s OFF WF: –	1 h/day for 10 sessions over 2 weeks	MDS‐UPDRS Cognitive function Self‐reported symptom improvement	Cognitive fluency performance declined Reduced Tremor and bradykinesin subgroup of responders	Multi‐days session; demonstrated safety and feasibility of taVNS	No significant overall motor and cognitive improvement; worsening of verbal fluency, variability in individual responses; need for biomarker validation and target engagement studies
Mondal et al., 2024^38^	Interventional, randomized, sham‐controlled, crossover (double blind) n = 33	tcVNS (GammaCore) Left neck VN C: manufacturer sham design	I: – PW: 100 μs F: 25 Hz DC: – WF: 5 kHz sine	2 × 120 s, 50–10 min intervals each day for 1 month	MDS‐UPDRS FOG‐Q TUG test Mattis dementia rating scale MMSE RBD questionnaire Circulating biomarkers (TNF‐a, IL‐6, IL‐10, BDNF)	Increased velocity, step length, rhythm. Reduced TNF‐α and oxidative stress	Objective gait measurement; biomarker study	Short term study; limited follow up; small sample size; limited molecular biomarker data; device‐related practical challenges
Zhang et al., 2024^37^	Interventional, randomized, sham‐controlled (double blind) n = 90	taVNS (tVNS501) Left cymba conchae ABVN C: earlobe	I: – PW: 200 μs F: 20 Hz/4 Hz DC: – WF: –	30 min day for 14 days	HAMA UPDRS I UPDRS III MoCA FAB HAMD‐24 VFT fNIRS (ΔHbO₂ in PFC)	Improved anxiety scales	Provided insights into the neural regulatory mechanisms underlying anxiety in PD; included an objective neuroimaging approach (fNIRS)	Short follow‐up period; small sample size; on drug condition; limited anxiety assessment tool; no language task included
Clinical studies in tremor/ET								
Clinical studies in tremor								
Marrosu et al., 2005^45^	Interventional, open label, single‐case	iVNS Cervical VN	I: 1 mA PW: 250 μs F: 10 Hz DC: 62 s ON/60 s OFF WF: –	18 months	EDSS KRS	Improvement in tremor severity	Longterm follow‐up	Single case report, uncontrolled, lack of objective measurement
Marrosu et al., 2007^46^	Interventional, open label n = 3	iVNS (Cyberonics) Cervical VN	I: 1.25 mA PW: 250 μs F: 10 Hz DC: 62 s ON/60 s OFF WF: –	26 months	KRS Water swallow test	Robust 67% reduction of postural upper limb and head tremor Improvement of dysphagia	Longterm follow‐up	Small sample size; uncontrolled; lack of objective measurement
Clinical studies in ET								
Handforth et al., 2003^47^	Interventional, open label n = 9	iVNS (Cyberonics) Cervical VN	I: 1.25 mA PW: 500 μs F: 20 Hz DC: 30 s ON/1.8 min OFF	4 weeks	TRS Accelerometry Videotape ratings	Improvement (50%) of hand tremor power No clinical change at blinded videorating	Objective tremor measurement (accelerometry); blinded videorating of tremor	Small sample size, uncontrolled
Marano et al., 2024^48^	Interventional, open label n = 17	tcVNS (GammaCore) Neck VN	I: – PW: 100 μs F: 25 Hz DC: – WF: 5 kHz sine	4 × 120 s	FTM scale Digital spiral analysis	Improvement of SWVI and spiral metrics No clinical changes at FTM	Objective spiral drawing evaluation (Trsper)	Small sample size, uncontrolled; no objective evaluation of tremor

Abbreviations: PD, Parkinson's disease; ET, essential tremor; VNS, vagus nerve stimulation; FOG, freezing of gait; Med, medication state; NA, not available; tcVNS, transcutaneous cervical vagus nerve stimulation; VN, vagus nerve; I, current intensity; PW, pulse width; F, frequency; DC, duty cycle; WF, waveform; UPDRS, Unified Parkinson's Disease Rating Scale; C, control/sham stimulation; taVNS, transcutaneous auricular vagus nerve stimulation; TUG, time up and go test; STN, subthalamic nucleus; APA, anticipatory postural adjustments; fMRI, functional magnetic resonance imaging; ALFF, amplitude of low‐frequency fluctuations; PreCG, precentral gyrus; PoCG, postcentral gyrus; SPL, superior parietal lobule; iVNS, surgical implanted vagus nerve stimulation; GSRS, gastrointestinal symptom rating scale; fNIRS, functional near‐infrared spectroscopy; MDS‐UPDRS, Movement Disorder Society‐Unified Parkinson's Disease Rating Scale; HAMA, Hamilton Anxiety Scale; HAMD, Hamilton Depression Scale; PFC, prefrontal cortex; FTM, Fahn‐Tolosa‐Marin.

Regarding atypical parkinsonism, taVNS (TENS‐200A, Hwato, Suzhou, China) has been reported in a single patient with MSA‐C, showing progressive long‐term improvements in both motor and non‐motor domains^42^ (further details are provided in Table S2).

### VNS in Tremor: Preclinical Experiments

Handforth and colleagues^43^ were the first to investigate iVNS in preclinical models of ET, adopting an open‐label experimental design. Their results demonstrated that iVNS significantly suppressed tremor in rats, with effects that persisted briefly after stimulation was discontinued.^43^ These findings were subsequently confirmed in a larger randomized, sham‐controlled, double‐blind study that reported a 40% reduction in tremor‐related movement power following iVNS.^44^ Both experiments were conducted using the Harmaline‐induced model of tremor.^43^, ^44^ The same research team conducted the first clinical trial of iVNS in ET patients (Table 1).

### VNS in Tremor: Clinical Experiments

The effects of VNS on tremor have been investigated in multiple sclerosis (MS) tremor and in ET. A case report and a small series of three MS patients, evaluated with the Klockgether rating scale for cerebellar symptoms, demonstrated robust and sustained improvement of postural tremor under iVNS (Cyberonics, Houston, TX).^45^, ^46^ Notably, low‐intensity stimulation delivered in short cycles (1 mA, 20 Hz, 250 μs, 60 seconds ON/60 seconds OFF) was effective in the long‐term management of MS‐related tremor. Later, Handforth et al.^47^ treated nine ET patients with iVNS (Cyberonics) for 4 weeks. Accelerometer analysis showed a 50.2% ± 31.8% reduction of postural tremor total power during stimulation; however, blinded videotape assessment failed to confirm clinically meaningful benefit and the trial program was subsequently discontinued.^47^ A recent open‐label study on 17 ET patients treated with tcVNS reported similar findings.^48^ After four consecutive 120 seconds stimulation trains with GammaCore, patients showed improvement on a digital spiral test, although no other objective changes were detected at Fahn‐Tolosa‐Marin (FTM) scale.^48^ The digital spiral assessment tool (i.e., Trsper, http://www.trsper.com) included validated tremor and ataxia metrics (i.e., spiral width variability index [SWVI]), raising the possibility that tcVNS may exert effects on cerebellar function.^47^, ^48^

### VNS in Other Movement Disorders

Evidence for VNS in MDs beyond PD and tremor is limited to case‐based reports in CD and TS. Kampusch and colleagues^49^, ^50^ reported the only documented use of taVNS (P‐Stim, Biegler, Austria) in CD (Table S2). In their first case study, the patient experienced subjective symptom improvement after 1 month of low frequency stimulation. In the subsequent follow‐up extended trial lasting 20 months, variable stimulation parameters and electromyography‐based assessment were used, demonstrating symptom alleviation through adaptive stimulation protocols specifically targeting Aβ‐fibers of the ABVN. In TS, Diamond and colleagues^51^ described a case of a patient with comorbid epilepsy who received iVNS. Blinded analysis of rush videos (Rush Tic Scale [RTS]) revealed a reduction in both motor and vocal tic severity, but no body distribution while on iVNS (vs. off iVNS), supporting a potential therapeutic effect in tics (Table S2).

## Discussion

**FIG. 2:**
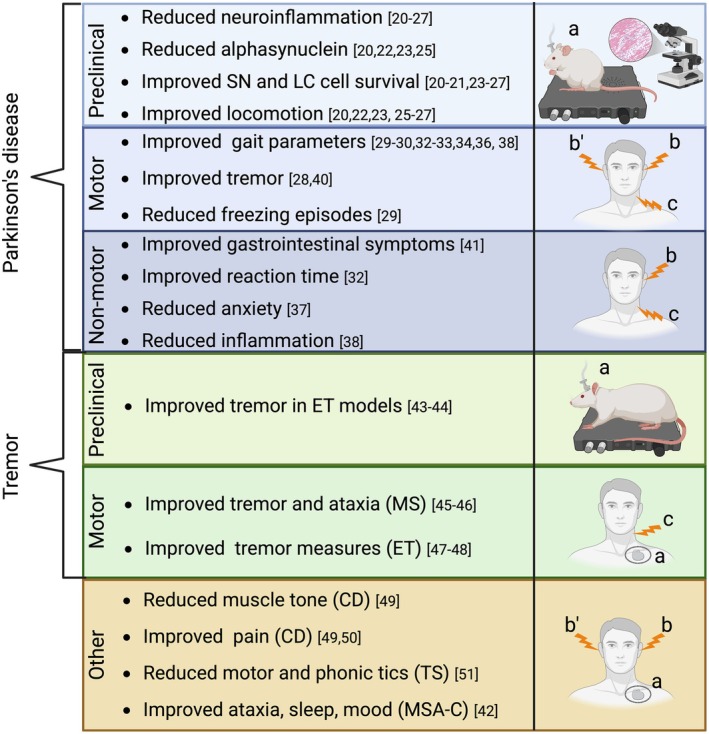
Effects of vagus nerve stimulation (VNS) in movement disorders. (a) invasive VNS (iVNS), (b) left transcutaneous auricular VNS (taVNS), (b’) right taVNS, (c) left transcutaneous cervical VNS (tcVNS). SN, substantia nigra; LC, locus coeruleus; ET, essential tremor; MS, multiple sclerosis; CD, Cervical Dystonia; TS, Tourette syndrome; MSA‐C, multiple system atrophy type C. Created in https://BioRender.com [Color figure can be viewed at wileyonlinelibrary.com]

VNS has emerged as a promising neuromodulation therapy for MDs, with the strongest evidence currently available in PD (Fig. 2). The studies included in this review suggest that VNS may ameliorate both motor and non‐motor symptoms, exert anti‐inflammatory and neuroprotective effects, and modulate large‐scale brain networks. However, the diversity of stimulation methods, small sample sizes, and lack of RCTs substantially limit the interpretability and generalizability of the findings.

### Anatomical and Functional Pathways of VNS Relevant to MDs

**FIG. 3:**
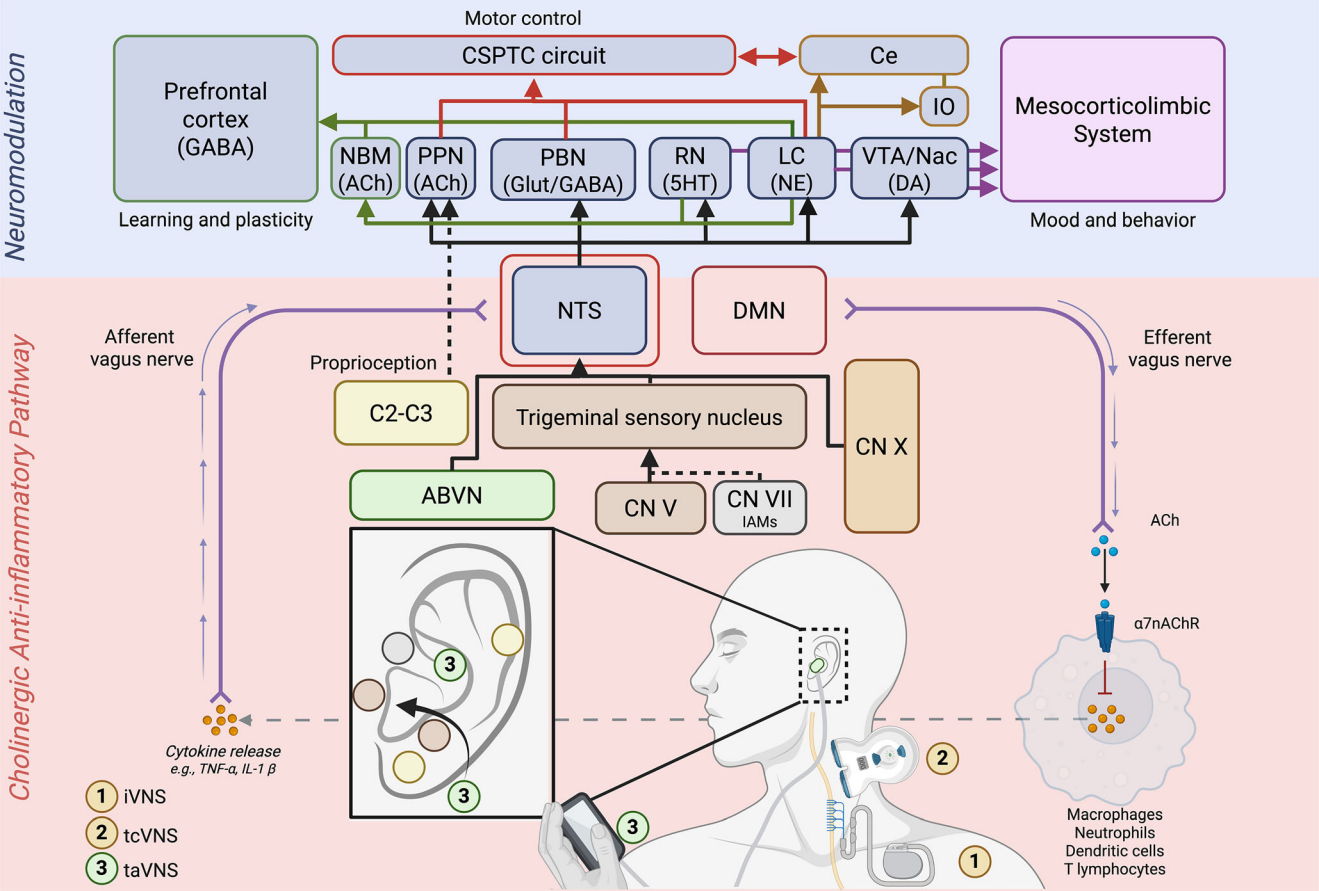
Schematic representation of VNS functional neuroanatomy and potential metabolic and neuromodulation effect. 5HT, serotonin; ABVN, auricular branch of the vagus nerve; ACh, acetylcholine; α7nAChR, α 7 nicotinic ACh receptor; C, cervical spine nucleus; Ce, cerebellum; CN, cranial nerve; CSPTC, cortico‐striatal‐pallidal‐thalamo‐cortical; DA, dopamine; DMN, dorsal motor nucleus of the vagus nerve; GABA, γ amino butirric acid; Glut, glutamate; IAMs, intrinsic auricular muscles; IO, inferior olivari nucleus; iVNS, invasive left cervical VNS; LC, locus coeruleus; NBM, nucleus basalis of Meynert; NE, norepinephrine; NTS, nucleus of the solitary tract; PBN, parabrachial nucleus; PPN, peduncolopontine nucleus; RN, raphe nucleus; taVNS, transcutaneous auricular VNS; tcVNS, transcutaneous cervical VNS; iVNS, invasive vagus nerve stimulation; VTA/Nac, ventral tegmental area/nucleus accumbens. Created in https://BioRender.com [Color figure can be viewed at wileyonlinelibrary.com]

The VN originates from a complex of motor and sensory nuclei in the medulla, including the nucleus ambiguous (NAmb, somato‐motor), the dorsal motor nucleus (DMN, viscero‐motor), and nuclei involved in visceral and somatic sensation. After exiting the skull through the jugular foramen, the VN gives rise to multiple branches, including the ABVN, which conveys somatic input from the ear.^52^ The VN comprises both afferent and efferent fibers. Afferents converge on the NTS, a key integrator of visceral sensory input, which then projects to subcortical and cortical areas involved in autonomic, emotional, and cognitive functions.^5^, ^52^ fMRI studies show that VNS modulates large‐scale brain networks, including the Central Autonomic Network (CAN), Default Mode Network (DMN), Salience Network (SN), and Ventromedial Affective Network (VAN).^53^, ^54^, ^55^, ^56^, ^57^ In patients treated for epilepsy or depression with left cervical VNS, blood‐oxygenated‐level‐dependent (BOLD) signal reductions have been observed in several regions, such as the cuneus, fusiform gyrus, anterior/mid‐cingulate, motor and sensory cortices, insula, temporal and frontal gyri, and superior parietal lobule.^58^ These effects unfold gradually over months,^58^ particularly in CAN‐related areas such as the anterior insula, medial prefrontal cortex, posterior cingulate, and hypothalamus.^59^ This broad connectivity provides a rationale for the diverse clinical effects of VNS, but also limits pathway specificity, making it difficult to link stimulation to specific clinical outcomes. The NTS is the primary central hub for vagal afferents, which sends widespread projections throughout the central nervous system (CNS) (Fig. 3).^5^ These include ascending pathways to monoaminergic nuclei such as the LC (NE), DRN (serotonergic, 5HT), and VTA (DA), with LC and DRN projecting to the NBM—the major source of cortical cholinergic innervation, crucial for attention, learning, and memory.^60^ NE and acetylcholine (ACh) are pivotal modulators of long‐lasting plasticity in both sensory and motor cortices.^2^, ^61^ Their engagement through VNS facilitates mechanisms such as long‐term potentiation (LTP) and depression (LTD), which underpin learning and adaptive motor control and support theoretically the adoption of VNS in post‐stroke rehabilitation.^62^ VTA/nucleus accumbens (Nac) VN mediated DA signaling is implicated in reward.^63^ In parallel, γ‐aminobutyric acid (GABA) plays a complementary role by regulating inhibitory tone and stabilizing cortical networks.^64^, ^65^ Notably, increased GABAergic activity following VNS has been demonstrated using double‐pulse transcranial magnetic stimulation (TMS) paradigms, specifically short‐interval cortical inhibition (SICI).^64^ This finding supports a role for VNS in restoring excitatory–inhibitory balance, which may be particularly relevant in MDs characterized by reduced inhibition, such as dystonia or tics. Finally, the NTS also projects to the PBN, which relays to thalamic nuclei (ventroposteromedial parvicellular, mediodorsal, midline and intralaminar nuclei) with downstream connection to the insula, PFC, amygdala and hippocampus. Finally, the vagal network influences cortico‐basal ganglia‐thalamic loop, with VNS facilitating dopamine release from the substantia nigra part compacta (via PBN).^66^

The efferent VN regulates autonomic and motor outputs to the viscera, as well as to the pharyngeal and laryngeal muscles. It also contributes to the regulation of immune and inflammatory responses through the cholinergic anti‐inflammatory pathway (CAP). In this reflex, the VN serves as the afferent branch, transmitting signals to the NTS and the DMN, which in turn promote ACh release from efferent VN fibers. This release activates α7 nicotinic ACh receptors (α7nAChRs) on immune cells, thereby influencing the Treg/Thelp balance^1^, ^17^ (Fig. 3). This duality underscores the VN's central role in autonomic homeostasis as the principal parasympathetic nerve.^52^

### Pathophysiological and Clinical Relevance of VNS in MDs

PD is the most extensively studied condition in MDs for both invasive and non‐invasive VNS. Preclinical studies, building on the seminal work of Farrand et al.,^20^, ^22^, ^23^ consistently demonstrated symptomatic and neuroprotective effects, mediated by vagal afferents, with evidence supporting a bilateral mechanism. Improved locomotor activity has been linked to the modulation of NE and DA networks, whereas antioxidant (e.g., increased glutathione), anti‐inflammatory actions (e.g., reduced TNF‐α and IL‐1β in the SN)—via CAP related mechanisms—and promotion of the BDNF–TrkB signaling likely account for the observed neuroprotection.^20^, ^21^, ^22^, ^23^, ^24^, ^25^, ^26^, ^27^ Together, preclinical studies support the view that taVNS may exert both neuroprotective/anti‐inflammatory effects, highlighting its promise as a disease‐modifying strategy for PD. Noteworthy, symptomatic and neuroprotective effects may arise from distinct mechanisms, with different stimulation frequencies preferential engaging each pathway in a specific disease model. For instance, high frequency (i.e., 300 Hz) microburst stimulation has been shown in animal PD models to elicit the strongest neuroprotective effects.^23^

The clinical translation of these experimental findings has been supported by small‐scale studies, with or without complementary instrumental assessments, such as gait analysis or neuroimaging, or circulating biomarkers. Early investigations suggested that 25 Hz tcVNS could improve gait impairment and possibly FOG in PD.^29^, ^30^ However, these studies were limited by small sample sizes and methodological heterogeneity. More recent work has shifted toward taVNS at varying stimulation frequencies, which may allow for more consistent engagement of afferent vagal pathways.^32^, ^33^, ^34^, ^35^, ^36^, ^37^, ^39^, ^40^ Importantly, the availability of manufacturer‐developed sham modalities has improved trial methodology, enabling more rigorous and better‐controlled study designs. Our group has provided evidence that 25 Hz tcVNS and taVNS can modulate brain oscillations relevant to PD, with motor improvements (e.g., gait speed) at least partly mediated by a reduction in STN β activity.^31^, ^33^ Notably, the effects of VNS appear to be partially independent of DA replacement therapy, as gait improvements were observed both in the *on* and *off* medication states.^32^, ^33^ VNS at 25 Hz has also been shown to modulate fMRI and fNIRS signals, similarly to what has been observed in epilepsy, and to reduce circulating inflammatory biomarkers across different studies.^36^, ^37^, ^38^ Nevertheless, different frequencies may play distinct roles even in treating motor symptoms in PD, with 25 Hz contributing more effectively to turning, whereas 100 Hz appears to benefit arm swing and APA.^34^ These frequency‐dependent effects may reflect selective engagement of distinct neural circuits and support the notion that tailored stimulation protocols could optimize therapeutic outcomes. Recent evidence indicates that cholinergic neuronal loss in the basal forebrain (NBM) and brainstem (PPN) contributes to gait decline.^67^ Enhancing cholinergic neurotransmission through the NTS‐PPN and NTS‐LC‐NBM pathways appears to be a key mechanism supporting gait improvement.

Although experimental findings from single‐session trials are encouraging, symptomatic changes and impact on patients' functionality were modest or barely measurable with clinical scales.^32^ Conclusive evidence from larger, well‐powered randomized trials is still lacking.^39^ In conclusion, although long‐term studies have reported improvements in non‐motor symptoms such as anxiety and gastrointestinal function,^37^ significant effects on motor endpoints have yet to be demonstrated.^39^

Evidence for tremor remains less robust. Results from treatment of MS‐related tremor are anedoctal,^45^, ^46^ whereas early trials of iVNS in ET suggested potential benefits, although clinical outcomes have been inconclusive.^47^ More recently, tcVNS studies have mirrored iVNS findings in ET, paving the way for further exploration of non‐invasive approaches to tremor management.^48^ Preclinical work using harmaline (tremorgenic Β carbolines inducing rhythmic burst‐firing activity in inferior olivary nuclei [IO]) supports this rationale, indicating that VNS may alleviate tremor by enhancing cortical GABAergic tone and modulating neuronal firing in the IO through LC‐NE, thereby disrupting the hypersynchronous activity between the IO, Purkinje cells (i.e., via climbing fibers), and cerebellar nuclei—a network implicated in ET pathophysiology.^43^, ^44^ Enhanced cortical GABA and inhibitory tone may also justify preliminary positive findings on dystonia and tics. Moreover, taVNS showed a frequency dependent propensity of modulating the cerebello‐thalamo‐cortical network, increasing cerebellar brain inhibition, possibly using the cerebellar network as an entry point to larger scale systems.^68^

However, for CD and TS, the available evidence is limited to isolated case reports, which describe modest or predominantly subjective improvements. Given that both disorders are characterized by reduced inhibition, maladaptive plasticity, and dysfunctional somatosensory processing within cortico‐striatum‐pallidal‐thalamic‐cortical loop,^69^, ^70^, ^71^ further studies investigating the potential of VNS are warranted. Overall, although PD and tremor currently represent the most promising indications for VNS, its role in other MDs remains highly exploratory.

### Methodological and Technical Considerations on VNS Modalities

iVNS is a neuromodulation technique in which surgically implanted internal pulse generator, connected to electrodes, deliver electrical impulses to the VN.^72^ Surgical implantation is generally safe, with a 25‐year study reporting an 8.6% complication rate^7^, ^73^ The procedure is typically performed on the left cervical VN to avoid arrhythmic complications related to cardiac vagal asymmetry.^16^, ^73^, ^74^ By the 1990s, implanted cuff electrodes were approved for drug‐resistant epilepsy. Although iVNS shows clinical promise, it remains underused because of its invasiveness and cost. This has driven the development of non‐invasive alternatives aimed at improving safety and accessibility.^75^, ^76^ Non‐invasive cervical VNS (tcVNS), FDA‐approved in 2017 for migraine (GammaCore),^76^ faces limitations because of the depth and anatomical complexity of the cervical VN, requiring higher stimulation intensity and posing a risk of off‐target effects despite the proprietary frequency‐modulated electrical stimulus (5 kHz sine wave, 1 ms, 25 Hz).^76^ In contrast, the ABVN, which is purely afferent and lies superficially at the inner tragus and cymba conchae, allows easier electrode placement, lower current demands, and a tailorable and safer stimulation^77^, ^78^ (Table S3). Commercially available devices, such as Parasym (London, UK), deliver a patented frequency‐modulated electrical stimulus—referred to as auricular vagus neuromodulation—via the tragus, and have demonstrated clinical feasibility.^79^, ^80^, ^81^, ^82^ However, the ABVN shares important anatomical connections with the trigeminal (CN V) and facial (CN VII) nerves, as well as with proprioceptive afferents from the cervical spinal ganglia (C2–C3). These inputs collectively innervate the external ear, including the tragus, antitragus, inferior concha (auriculotemporal nerve, CN V), the intrinsic auricular muscles (helices minor, tragus and antitragus muscles; CN VII), and helix/earlobe (great auricular nerve, C2–C3)^83^ (Fig. 3). Notably, trigeminal afferents also provide a non‐vagal route to the NTS, raising concerns about the anatomical specificity of auricular stimulation techniques such as taVNS and, to some extent, intrinsic auricular muscle zone stimulation.^83^, ^84^, ^85^ Although taVNS offers clear advantages in safety, tolerability, accessibility^86^, ^87^, ^88^, ^89^ concerns remain regarding the overlap between vagal and non‐vagal inputs, which raises skepticism about device efficacy. However, converging evidence from functional imaging and neurophysiological studies demonstrates that taVNS does engage vagal pathways,^64^, ^90^ indicating that although non‐vagal co‐activation may limit anatomical specificity, it does not preclude clinically meaningful effects.

Effective VNS depends not only on the stimulation site, but also on precise parameters, including intensity, pulse width, frequency, and waveform. Most taVNS studies have used fixed intensities, although some protocols adjust stimulation based on individual perception.^91^ Husley and colleagues^92^ demonstrated that LC activation increases with higher frequency and pulse width; however, cognitive effects follow an inverted U‐shaped pattern, with moderate stimulation level yielding the most beneficial outcomes.^93^, ^94^

Notably, the effects on NE and DA appear to be optimized by ON–OFF cycling at intermediate frequencies (20–30 Hz),^95^ consistent with rodent studies indicating that periodic waveforms most effectively enhance LC firing and NE release. In contrast, pro‐cognitive effects of taVNS have been observed at lower frequencies (~5 Hz), whereas cortical GABAergic signaling is preferentially facilitated at intermediate to high frequencies (20–100 Hz).^95^ More recently, very high‐frequency stimulation (≈300 Hz) delivered in bursts has been explored for its potential disease‐modifying properties in PD models.^23^

No consensus exists on the optimal frequency, and most protocols use 20 to 30 Hz.^96^ A 2021 review emphasized that current intensity and pulse width a are more critical for effective VN activation and neurotransmitter release than frequency.^96^, ^97^ Importantly, stronger stimulation does not necessarily translate into better outcomes: both human and animal studies indicate that moderate stimulation achieves the most favorable cognitive effect.^93^, ^94^ Early iVNS systems used long off‐cycles (e.g., 30 seconds ON/5 minutes OFF) to minimize nerve fatigue and preserve battery life. In contrast, more recent studies suggest that pairing stimulation bursts with behavioral tasks yields stronger neuroplastic effects than fixed duty cycles.^96^ This will support the adoption of VNS in rehabilitation of neurological diseases.^98^ Compared to implanted systems, tcVNS and taVNS offers key advantages, including non‐invasive delivery, absence surgical risks, and fewer side effects.

Different VNS modalities offer complementary strengths and limitations (Table S3). Non‐invasive VNS has enabled the clinical translation of preclinical iVNS findings, whereas human application of iVNS itself remains limited to two small studies in tremor and the few small size or single case reports in PD, MS‐tremor, and TS.

All tcVNS experiments were performed with the GammaCore device (n = 6). Among these, all controlled studies (n = 3) used sham stimulators specifically engineered to avoid activation of cervical VN fibers. Eleven taVNS (8 PD, 1 MSA, 2 CD) studies used seven different devices, three of which were custom‐built, with stimulation sites including the left tragus (n = 3), the left cymba conchae (n = 7), and bilateral tragus and cymba conchae (n = 1). Seven taVNS studies incorporated control conditions, consisting of left earlobe stimulation in six cases and stimulation of the left tail of the helix in one case. Both control sites are located outside the ABVN territory and are primarily innervated by the great auricular nerve (C2–C3), with a further potential contribution by CN V fibers.^83^

To date, no modality has demonstrated clear experimental superiority. Head‐to‐head comparisons under standardized protocols are needed to establish their relative therapeutic value. Methodological issues remain central. Across studies, stimulation protocols vary widely (site, frequency, intensity, and duty cycle), and sham conditions are often suboptimal (e.g., earlobe/helix stimulation), contributing to heterogeneity of results. Both taVNS and tcVNS demonstrated similar trends in their results. Although both modalities activate the CAN, only iVNS and tcVNS are capable of recruiting both afferent and efferent fibers. Notably, the only RCT addressing autonomic dysfunction (i.e., gastrointestinal symptoms) was conducted using tcVNS.

## Future Directions and Conclusions

In summary, VNS represents a promising multimodal neuromodulation strategy for MDs, with the strongest evidence emerging in PD and preliminary, but less consistent data in tremor, dystonia, and tics. Large‐scale controlled trials are essential before VNS can be fully integrated into the therapeutic landscape for MDs. Preclinical studies support both symptomatic and neuroprotective mechanisms, but clinical translation is still constrained by small samples, heterogeneous protocols, and lack of adequately powered RCTs. A promising avenue is closed‐loop VNS, which integrates real‐time biosensing and adaptive stimulation, to deliver tailored or paired therapy.^99^, ^100^, ^101^ However, future research should prioritize standardized stimulation parameters and mechanistic studies including neurophysiology,^102^, ^103^, ^104^, ^105^ and neuroimaging to clarify which pathways mediate specific clinical effects.

## Author Roles

(1) Research project: A. Conception, B. Organization, C. Execution; (2) Statistical Analysis: A. Design, B. Execution, C. Review and Critique; (3) Manuscript: A. Writing of the First Draft, B. Review and Critique.

F.P.: 1B, 1C, 3A

M.M.O.: 3A

E.B.: 3A

G.V.: 3B

G.A.: 3A

R.A.R.: 3B

F.C.: 3B

V.G.M.: 3AB

V.D.L.: 3B

M.M.: 1A, 1B, 1C, 3A, 3B

## Financial Disclosures

M.M. received travel grants and/or speaking honoraria from AbbVie, Medtronic, Ipsen, Sanofi, PIAM, and Zambon. E.B. is medical director of Parasym. Other authors deny any financial disclosure for the preceding 12 months.

## Supporting information


**Table S1.** Risk of Bias Assessment of included Randomized Controlled Trial using Cochrane Risk‐of‐Bias tool (RoB 2).

**Table S2.** Clinical studies in other movement disorders.

**Table S3.** Comparison between techniques.

## Data Availability

Data sharing not applicable to this article as no datasets were generated or analysed during the current study.
