# Supplementary material for: Vagus Nerve Stimulation in Movement Disorders, from Principles to a Systematic Review of Evidence
Source: Mov Disord. 2025 Sep 30;40(12):2559–77. doi: 10.1002/mds.70044 (PMC12710210; doi:10.1002/mds.70044)
Supplement: Supplementary file 1 — Table S1. Risk of Bias Assessment of included Randomized Controlled Trial using Cochrane Risk‐of‐Bias tool (RoB 2). [file MDS-40-2559-s003.docx]

**Supplementary table 1.** Risk of Bias Assessment of included Randomized Controlled Trial using Cochrane Risk-of-Bias tool (RoB 2).

| **Study** | **Risk of bias arising from the randomization** | **Risk of bias due to deviations from the intended interventions** | **Missing outcome data** | **Risk of bias in measurement of the outcome** | **Risk of bias in selection of the reported result** | **Overall risk of bias** |
| --- | --- | --- | --- | --- | --- | --- |
| **Marano M. et al., 2022** | Some concerns | Low risk | Low risk | Some concerns | Some concerns | Some concerns |
| **Kaut O. et al., 2019** | Some  concerns | Low risk | Some concerns | Some concerns | High risk | High risk |
| **Zhang H. et al., 2023** | Low risk | Low risk | Low risk | Some concerns | Some concerns | Some concerns |
| **Lench D.H. et al., 2023** | Some concerns | Low risk | Some concerns | Some concerns | Some concerns | Some concerns |
| **Mondal B. et al., 2024** | Some concerns | Low risk | Some concerns | Low risk | High risk | High risk |
| **Van Midden V. et al., 2024** | Some concerns | Low risk | Some concerns | Low risk | Some concerns | Some concerns |
| **Marano M. et al., 2024** | Some concerns | Low risk | Low risk | Some concerns | Some concerns | Some concerns |
| **Zhang H. et al., 2024** | Low risk | Low risk | Low risk | Some concerns | Some concerns | Some concerns |
| **Fu C. et al., 2024** | Some concerns | Low risk | Low risk | High risk | Some concerns | High risk |
| **Morris R. et al., 2019** | Some  concerns | Low risk | Some concerns | Low risk | Some concerns | Some concerns |

Note: “Low” = low risk of bias; “Some concerns” = potential risk of bias that could affect the outcome; “High” = high risk of bias likely to influence the results.
