# Supplementary material for: Vagus Nerve Stimulation in Movement Disorders, from Principles to a Systematic Review of Evidence
Source: Mov Disord. 2025 Sep 30;40(12):2559–77. doi: 10.1002/mds.70044 (PMC12710210; doi:10.1002/mds.70044)
Supplement: Supplementary file 2 — Table S2. Clinical studies in other movement disorders. [file MDS-40-2559-s002.docx]

**Supplementary table 2.** Clinical studies in other movement disorders.

| **Clinical studies in parkinsonisms** | | |  |  |  |  |  |  |
| --- | --- | --- | --- | --- | --- | --- | --- | --- |
| **Author/ Year** | **Study design** | **Technique** | **Parameters** | **Time of stimulation** | **Outcomes** | **Main results** | **Strengths** | **Limitations** |
| Wang et al., 2025 [42] | Case-report | taVNS  TENS-200A  Left Cymba conchae | - I: ≤50 mA - PW: 200 µs - F: 20 Hz (7 s) / 4 Hz (3 s) - DC: NA - WF: biphasic | 40 min, 20 times a month for 12 months | - SARA - UMSARS - PSQI - HAMA - HAMD | Improvements of ataxia and non-motor symptoms | First study who explore effect of taVNS on MSA-C; No adverse effect | Single-case study, lacked objective parameters and markers |
| **Clinical studies in dystonia** | | |  |  |  |  |  |  |
| **Author/ Year** | **Study design** | **Technique** | **Parameters** | **Time of stimulation** | **Outcomes** | **Main results** | **Strengths** | **Limitations** |
| Kampusch et al., 2013 [49] | Single-case | taVNS  P-Stim  Cymba conchae | - I: 4V - PW: 100 µs - F: 1 Hz - DC: 3 hours ON 3 hours OFF | 1 month | - EMG | - Subjective improvement in motility in a patient with CD | Provides preliminary evidence that taVNS may be a viable treatment for CD | Single case study; need for more comprehensive functional assessments; no long-term follow-up​ |
| Kampusch et al., 2015 [50] | Single-case | taVNS  P-Stim  Cymba conchae | - I: 0-2 V - PW: 500- 1000 µs - F: 1-100 Hz - DC: 110 min ON 15 min OFF | 20 months | - VAS - sEMG symptoms - sympathovagal balance | - Provided experimental evidence of significant reduction in muscle tone and autonomic regulation - pain perception decreased, and improvement in dystonic symptoms was achieved | Potential long-term benefits of taVNS in dystonia-related pain management; potential of taVNS as an effective neuromodulatory therapy for dystonia-related muscle hyperactivity | Single case study; uncontrolled. |
| **Clinical studies in Tourette syndrome** | | |  |  |  |  |  |  |
| **Author/ Year** | **Study design** | **Technique** | **Parameters** | **Time of stimulation** | **Outcomes** | **Main results** | **Strengths** | **Limitations** |
| Diamond et al., 2006 [51] | Single-case  (Blinded evaluation) | iVNS | - I: 1.25 mA - PW: 500 ms - F: 30 Hz - DC: 30 sec ON, 3 min OFF | 10 months | - Rush tic scale videotape | - Improvements in motor tic and phonic tic frequency and severity of phonic tics   no changes in the number of body parts affected or severity of motor tics | Overall tic burden improved suggesting a moderate therapeutic benefit; subjective improvements were validated with blinded video assessments; marked reduction in premonitory sensations | Single case study, lack of prospective longitudinal data; optimal stimulation settings for tic reduction remain unknown |

I, current intensity; PW, pulse width; F: frequency; DC: duty cycle; WF: waveform; C: control/sham stimulation; iVNS, surgical implanted vagus nerve stimulation; taVNS, transcutaneous auricular vagus nerve stimulation.
