# Supplementary material for: Vagus Nerve Stimulation in Movement Disorders, from Principles to a Systematic Review of Evidence
Source: Mov Disord. 2025 Sep 30;40(12):2559–77. doi: 10.1002/mds.70044 (PMC12710210; doi:10.1002/mds.70044)
Supplement: Supplementary file 3 — Table S3. Comparison between techniques. [file MDS-40-2559-s001.docx]

|  | **iVNS (invasive cervical)** | **tcVNS (transcutaneous cervical)** | **taVNS (transauricular cervical)** |
| --- | --- | --- | --- |
| Delivery methods | Implanted cuff electrode around left cervical VN | Handheld device over cervical VN | Electrodes on tragus/cymba conchae (ABVN) |
| Safety/tolerability | Surgical risks, hoarseness, cough | Efferent engagement  Ad hoc self-administration | Afferent selectivity  Ear-clips/ear-plugs connected to cable and portable stimulator |
| Target engagement | High (direct VN placement)  Afferent/efferent | Moderate (deep location of VN)  Afferent/efferent | Moderate (interindividual variability)  Afferent only |
| Target selectivity | Moderate (afferent and efferent VN) | Modest (afferent and efferent VN, near tissues and autonomic fibers) | Moderate (afferent VN)  Risk of trigeminal involvement |
| Typical parameters | 20-30Hz  250-500 us  30s ON/3-5 min OFF | 25 Hz  100 us  120 s stimulation | 10-25 Hz  200-500 us  ON/OFF cycle depending on manufacturer/design |
| Evidence in MDs | Strong preclinical  Few case report/series in tremor and MDs | Small OL studies and RCTs in PD  OL trials in ET | Growing clinical data in PD  Case reports in CD |
| Strength | Target engagement | Reliability | Low risk  Stimulation tailoring |
| Limitations | Cost, surgery, limited evidence in MDs | Risk of off-target effects  Requires high current intensity  Efferent engagement | Anatomical overlap with non-VN afferents  Device heterogeneity Protocol heterogeneity |

**Supplementary table 3.** Comparison between techniques.
